# Assessment of OCT and Angio-OCT Parameters in Keratoconus Patients with and without Penetrating Keratoplasty

**DOI:** 10.3390/jcm13175111

**Published:** 2024-08-28

**Authors:** Anna Maria Gadamer, Piotr Miklaszewski, Dominika Janiszewska-Bil, Anita Lyssek-Boroń, Dariusz Dobrowolski, Edward Wylęgała, Beniamin Oskar Grabarek, Aleksandra Kiełbasińska, Katarzyna Krysik

**Affiliations:** 1 Department of Ophthalmology, St. Barbara Hospital, Trauma Centre, 41-200 Sosnowiec, Poland; piotrmiklaszewski94@gmail.com (P.M.); dominika.bjaniszewska@gmail.com (D.J.-B.); anitaboron3@gmail.com (A.L.-B.); dardobmd@wp.pl (D.D.); kkrysik@gmail.com (K.K.); 2 Department of Ophthalmology, Faculty of Medicine, Academy of Silesia, 40-555 Katowice, Poland; 3 Collegium Medicum, WSB University, 41-300 Dabrowa Gornicza, Poland; bgrabarek7@gmail.com (B.O.G.); aleksandrakielbasinska96@gmail.com (A.K.); 4 Department of Ophthalmology, District Railway Hospital, 40-760 Katowice, Poland; wylegala@gmail.com; 5 Department of Ophthalmology, Faculty of Medicine, Medical University of Silesia, 40-555 Katowice, Poland

**Keywords:** keratoconus, penetrating keratoplasty, optical coherence tomography, angio-OCT, radial peripapillary capillaries, retinal nerve fiber layer, ganglion cell complex, central retinal thickness

## Abstract

**Background/Objectives:** Keratoconus (KC) is a bilateral eye disease characterized by corneal thinning and cone-like deformation, leading to visual impairment. This study evaluated the radial peripapillary capillaries (RPCs) in keratoconus patients with and without penetrating keratoplasty (PKP) using OCT and angio-OCT, comparing the results to a control group. **Methods:** This retrospective study included 149 eyes, 97 from patients who underwent PKP between January 2018 and February 2023 and 52 from patients who did not undergo PKP. The control group comprised 72 patients (144 eyes) who were healthy volunteers. Measurements included the best corrected visual acuity (BCVA), the intraocular pressure (IOP), slit-lamp biomicroscopy, a fundus examination, and corneal topography, as well as OCT and angio-OCT assessments of the RPCs, retinal nerve fiber layer (RNFL), ganglion cell complex (GCC), and central retinal thickness (CRT). Statistical analyses were performed using Student’s *t*-test and Pearson’s correlation coefficient. **Results:** The RNFL was significantly thinner in KC eyes after PKP compared to control eyes (*p* < 0.001), and the CRT was significantly thicker in KC eyes after PKP compared to control eyes (*p* = 0.003). However, the GCC was similar across the groups (*p* = 0.0885). Additionally, RPCs inside the disc were significantly reduced in KC eyes after PKP compared to control eyes (*p* < 0.0001). A significant positive correlation was found between RPC whole vessel density and RNFL thickness as measured via angio-OCT (r = 0.308, *p* < 0.0001). **Conclusions:** This study found that the RPC density inside the disc is significantly reduced in keratoconus patients after penetrating keratoplasty, highlighting RPCs inside the disc as a potential diagnostic tool for further assessment of keratoconus.

## 1. Introduction

Keratoconus (KC) is an asymmetric bilateral disease of the eye, where the cornea becomes cone-like due to structural thinning [1]. The disease leads to deterioration in vision, myopia, irregular astigmatism, and secondary corneal scarring [2]. Other corneal diseases that may imitate keratoconus include ectatic disorders such as pellucid marginal degeneration or keratoglobus, posterior keratoconus, and Fuchs or Terrien marginal degeneration [3].

The global prevalence of KC is estimated to be 1 in 2000 individuals, with a tendency to manifest in the second or third decade of life [4]. While the exact etiology of KC remains unclear, it has been associated with factors such as eye rubbing, atopic diseases, environmental influences, contact lens wearing, genetic predisposition, connective tissue disorders [5], and tapetoretinal degenerations, among others [6].

Improvements in visual acuity in keratoconus patients begin with spectacles or contact lenses. Further options such as collagen cross-linking, intracorneal rings implantation, surface-based keratorefractive laser procedures, phakic intraocular lens (IOL) implantation, and cataract extraction with toric in-the-bag IOL implantation are also available. Surgical treatment is necessary in the late stages of the disease, such as penetrating keratoplasty (PKP) or deep anterior lamellar keratoplasty (DALK) [7]. PKP is one of the most commonly performed surgical procedures for advanced KC. PKP involves the full-thickness transplantation of donor corneal tissue to replace the diseased cornea, thereby restoring corneal integrity and improving visual acuity. Despite its effectiveness, PKP is associated with several potential complications, including graft rejection, astigmatism, and the need for long-term postoperative care. The success of PKP in KC patients is often determined by careful postoperative monitoring and management, which is crucial for identifying early signs of graft failure or other complications. As such, PKP remains a vital surgical option for those with severe KC, offering a pathway to visual rehabilitation for patients whose condition has advanced beyond the reach of less invasive treatments [8,9,10,11].

Optical coherence tomography (OCT) and its angiographic extension, angio-OCT, have revolutionized the imaging and diagnostic capabilities in ophthalmology. OCT provides detailed cross-sectional images of the retina, allowing precise measurements of retinal layers, including the retinal nerve fiber layer (RNFL), ganglion cell complex (GCC), and central retinal thickness (CRT) [12,13]. Angio-OCT, on the other hand, offers a non-invasive method to visualize and quantify retinal and choroidal microvasculature by capturing dynamic changes in blood flow [13,14,15]. In the context of KC patients who have undergone PKP, these imaging modalities are invaluable. They not only facilitate the assessment of structural integrity and graft health post-surgery but also enable the detection of subtle vascular changes that could indicate early graft rejection or other complications. This imaging technique is increasingly used to investigate various diseases, especially by measuring the radial peripapillary capillaries (RPCs). Nowadays, RPCs are measured in glaucoma, myopia, multiple sclerosis, neurodegenerative diseases, and many others [16,17,18]. While the current body of literature highlights the potential of OCT in the early detection and monitoring of KC, further studies are required to establish its definitive role in the diagnostic algorithm. Specifically, more research is needed to validate the sensitivity and specificity of OCT in differentiating KC from other corneal ectasias and to determine the most effective OCT parameters for diagnosis. The role of angio-OCT is particularly promising in monitoring RPC density, which has emerged as a potential biomarker for assessing disease progression and postoperative outcomes in KC patients [19,20,21]. By providing comprehensive data on both the structural and vascular aspects of the retina, OCT and angio-OCT help in the early identification of complications, thereby guiding timely therapeutic interventions and improving the overall prognosis for KC patients following PKP [19,20,21].

This study evaluated the RPCs in keratoconus patients with and without PKP using OCT and angio-OCT, comparing the results to a control group.

## 2. Materials and Methods

### 2.1. Study Design

This retrospective study was conducted with the approval of the Ethics Committee (Nr 25/KB/AŚ/04/2024), obtained on 3 April 2024, and in accordance with the principles of the Declaration of Helsinki. Written informed consent was obtained from all participants. Patients received a leaflet explaining the nature of the study.

The study was performed at the Ophthalmology Department of Saint Barbara Hospital, Trauma Center, Sosnowiec, Poland. Our analysis included 149 eyes from 97 KC patients who underwent PKP between January 2018 and February 2023, as well as 52 KC patients who did not undergo PKP. The analyzed group included 78 patients diagnosed with keratoconus who underwent penetrating keratoplasty. Sixty-nine patients had keratoconus in both eyes, and seven had unilateral presentation. Nineteen patients underwent PKP in both eyes.

The control group comprised 144 eyes from healthy volunteers. There was no statistically significant difference with respect to sex or age between the study group, which included patients with KC who had or had not undergone PKP, and the control group (*p* > 0.05).

The operations were performed by two experienced ophthalmic surgeons. All patients underwent complete ocular examination, including measurements of the best corrected visual acuity (BCVA) using the Snellen chart, IOP measurements with an i-care tonometer, slit-lamp biomicroscopy, and a fundus examination with dilated pupils (1% Tropicamide WZF, Polpharma, Warsaw, Poland). Moreover, participants underwent corneal topography, both OCT and angio-OCT. All measurements were obtained by one researcher. Devices were placed in a darkened room to minimize external factors.

Before the ophthalmological examination, participants wearing contact lenses were required to stop wearing them for a minimum period of 2 weeks (soft contact lenses) or 3 weeks (rigid gas-permeable contact lenses, scleral contact lenses, hybrid contact lenses). All patients in the study group underwent examination 12–14 months after corneal transplantation.

### 2.2. Subjects

#### 2.2.1. Characteristics of the KC Patients Who Did Not Undergo PKP

This group of subjects consisted of 52 eyes (26 left eyes and 26 right eyes). Fifteen eyes were those of females (28.85%), where the mean age was 36.63 ± 11.23 years, and thirty-seven eyes belonged to males (71.15%), where the mean age was 35.18 ± 10.87 years. The inclusion criteria were as follows: written informed consent to participate in the research, age above 18 years, KC patient without PKP, and Polish Caucasian. The exclusion criteria were the following: refusal to participate in the study; age under 18 years; no diagnosed keratoconus; documented cognitive impairment; pregnancy; any corneal pathology other than keratoconus; ophthalmological diseases such as glaucoma, retinal, choroidal, or optic nerve diseases; or cardiovascular disease.

#### 2.2.2. Characteristics of the KC Patients Who Underwent PKP

This group of subjects consisted of 97 eyes that underwent PKP (33 left eyes, 26 right eyes, 38 both of a participant’s eyes—left and right). Twenty-three were from females (23.71%), where the mean age was 37.03 ± 11.01 years, and seventy-four were from males (76.29%), where the mean age was 35.34 ± 9.58 years. The inclusion criteria were as follows: informed consent from the participant to participate in research, age above 18 years, KC eye after PKP, and Polish Caucasian. The exclusion criteria were the following: refusal to participate in the study; age under 18 years; no diagnosed KC; other type of corneal transplantation; pregnancy; any corneal pathology other than keratoconus; ophthalmological diseases such as glaucoma, retinal, choroidal, or optic nerve diseases; or cardiovascular disease.

#### 2.2.3. Characteristics of the Control Group

The control group were healthy volunteers without any ophthalmological or systemic disease. The participants were adults of both sexes. Only Polish Caucasians without systemic diseases were included in the research to minimize confounding factors. The control group consisted of 72 patients (144 eyes). These included 20 females (40 eyes, representing 30.77%), where the mean age was 36.53 ± 11.80 years, and 52 males (104 eyes, representing 69.23%), where the mean age was 36.07 ± 9.62 years. The inclusion criteria were the following: informed consent to participate in research, age above 18 years, no ophthalmological or general diseases, and Polish Caucasian. The exclusion criteria were refusal to participate in the study, age under 18 years, documented cognitive impairment, or pregnancy.

### 2.3. Optical Coherence Tomography

In this study, we utilized the SOLIX spectral domain optical coherence tomography (SD-OCT) system (Optovue, Fremont, CA, USA), which operates at 120,000 A-scans per second and employs the split spectrum amplitude-decorrelation angiography algorithm. This algorithm differentiates between static and non-static tissue (e.g., blood flow), enabling the visualization of vessels by calculating the decorrelation signal amplitude from consecutive B-scans at the same retinal location. The SOLIX system is designed for in vivo cross-sectional and three-dimensional imaging and measurement of both anterior and posterior ocular structures. It includes an integrated reference database for comparing measurements to those of known normal subjects. The AngioVue software 2.1. (Optovue AngioVue^®^, Optovue, Inc., Freemont, CA, USA) includes features that aid in visualizing vascular structures of the retina and choroid in both normal subjects and those with glaucoma or retinal diseases [22,23,24].

For this study, we employed AngioAnalytics™ to measure the retinal vessel density, foveal avascular zone (FAZ), flow area, non-flow area, and retinal layer thickness, using 6.4 mm AngioVue Retina scans (Optovue, Inc., Freemont, CA, USA). The scan area was 6.4 mm × 6.4 mm for AngioVue Retina and Retina Cube, and 6 mm × 6 mm for AngioVue Disc and Disc Cube. AngioVue Disc measurements included the RPC density and RNFL thickness for the 4.5 mm AngioVue Disc. The vessel density analysis involved calculating the percentage of the area occupied by angio-OCT-detected vasculature for the RPC slab from the internal limiting membrane (ILM) to the RNFL. The peripapillary RNFL thickness was measured specifically for the RNFL (ILM to RNFL). The small vessel density was measured using a large vessel mask with a threshold of ≥3 pixels (approximately ≥35 μm for the 6.0 mm AngioVue Disc scans). After the mask was applied, only the small vessel density was measured.

For image selection, only OCT and angio-OCT images with a high signal strength (≥6/10) were accepted for inclusion in the study. Images with a signal strength below this threshold were excluded to ensure the accuracy and reliability of the measurements. This criterion was applied uniformly across all scans to maintain consistency and data integrity. We investigated the average RNFL thickness, average ganglion cell complex (GCC), and central retinal thickness (CRT) in the fovea using both OCT and angio-OCT in different groups of keratoconus (KC) patients. Additionally, we analyzed the RPC parameters in detail. For RPC density, we imaged all vessels (small and large), measuring the average, inferior, and superior peripapillary small vessel density and the small vessel density inside the disc. The results were then compared to those from control eyes.

The images were analyzed by two experienced ophthalmic surgeons. The Kappa value was calculated to determine the level of agreement between the raters assessing OCT and angio-OCT images. The Kappa statistic for this study was found to be 0.90, which indicates almost perfect agreement among the raters. This level of agreement supports the reliability of the assessment process used in this study.

### 2.4. Statistical Analysis

Statistical analysis was performed using Statistica software, version 13 (StatSoft, Cracow, Poland). The data were first tested for normality using the Shapiro–Wilk test. For normally distributed data, one-way analysis of variance (ANOVA) was used to compare mean values across the groups. When the ANOVA *p*-value was less than or equal to 0.05, indicating a statistically significant difference among groups, a post hoc analysis was performed using Tukey’s honest significant difference (HSD) test to identify which specific groups differed from each other.

However, if the ANOVA *p*-value was greater than 0.05, indicating no statistically significant difference among the groups, no post hoc test was conducted. This approach ensured that only meaningful comparisons were made, reducing the risk of type I errors associated with multiple testing. Descriptive statistics are given as the mean, standard deviation, median, and 95% confidence interval. To measure the linear relationship between RNFL and RPCs, Pearson’s product moment correlation coefficient was used. To ensure the consistency and reliability of the measurements, inter-rater reliability was assessed using the Kappa statistic. A *p* value of < 0.05 was considered statistically significant.

## 3. Results

### 3.1. Evaluation of OCT and Angio-OCT in Particular Groups

Table 1 shows the results for average RNFL, average GCC, and CRT obtained with OCT and angio-OCT for each group.

For the RNFL measured using OCT, Group I had a mean thickness of 88.96 ± 13.02 µm, while Group II had a similar mean thickness of 88.33 ± 10.95 µm. Group III, the control group, showed a slightly higher mean RNFL thickness of 92.42 ± 7.67 µm. The analysis of variance (ANOVA) revealed a significant difference among the groups (*p* = 0.0037). Post hoc Tukey’s test identified a significant difference between Group II and Group III (*p* = 0.0115), indicating that the RNFL thickness in the control eyes was significantly higher than that in keratoconic eyes after PKP.

When the GCC was examined using OCT, Group I had a mean thickness of 101.81 ± 8.77 µm, Group II had a mean thickness of 100.78 ± 9.40 µm, and Group III showed a slightly higher mean thickness of 102.81 ± 7.52 µm. However, the ANOVA did not show a significant difference among the groups (*p* = 0.2027); therefore, no post hoc analysis was performed.

For the central retinal thickness (CRT), Group I had a mean CRT of 269.85 ± 18.99 µm, Group II had a mean CRT of 267.90 ± 19.21 µm, and Group III had a lower mean CRT of 260.00 ± 21.14 µm. The ANOVA indicated a significant difference among the groups (*p* = 0.0016), and post hoc Tukey’s test revealed significant differences between Group I and Group III (*p* = 0.0175) and between Group I and Group II (*p* = 0.0340). This suggests that the CRT in keratoconic eyes without PKP was significantly thicker compared to that in control eyes.

The results for angio-OCT parameters showed similar trends. For the RNFL, Group I had a mean thickness of 88.96 ± 13.45 µm, Group II had a mean thickness of 88.60 ± 11.18 µm, and Group III had a higher mean thickness of 92.85 ± 7.92 µm. The ANOVA again showed a significant difference among the groups (*p* = 0.0028), with post hoc Tukey’s test confirming a significant difference between Group II and Group III (*p* = 0.0103), indicating that RNFL thickness was significantly greater in control eyes compared to keratoconic eyes after PKP.

For the GCC measured with angio-OCT, the mean thickness was 101.92 ± 9.30 µm in Group I, 101.21 ± 9.23 µm in Group II, and 103.40 ± 7.70 µm in Group III. The ANOVA did not reveal significant differences among these groups (*p* = 0.1351); therefore, no further post hoc analysis was conducted.

Regarding the CRT, Group I had a mean of 270.56 ± 20.65 µm, Group II had a mean of 266.89 ± 19.17 µm, and Group III had a mean of 262.09 ± 20.47 µm. The ANOVA indicated a significant difference (*p* = 0.0209), with post hoc Tukey’s test showing a significant difference between Group I and Group III (*p* = 0.0248), suggesting that the CRT in keratoconic eyes without PKP was significantly thicker than that in control eyes.

In summary, the analysis revealed that keratoconic eyes, particularly those without PKP, exhibited distinct differences in the RNFL and CRT compared to control eyes. These differences were especially notable in the RNFL thickness and CRT, with keratoconic eyes generally showing greater thickness in these parameters, a trend observed in both OCT and angio-OCT measurements. However, no significant differences were observed in GCC thickness among the groups (*p* > 0.05).

### 3.2. Evaluation of the RPC Parameters in Particular Groups

The results presented in Table 2 highlight the RPC parameters across the three groups: Group I (keratoconic eyes without PKP), Group II (keratoconic eyes after PKP), and Group III (control eyes).

For the RPCs inside the disk, Group I exhibited a mean value of 42.23 ± 7.22 µm, Group II had a higher mean of 44.36 ± 7.65 µm, while Group III (control eyes) showed a slightly lower mean of 41.09 ± 6.75 µm. The ANOVA indicated a highly significant difference among the groups (*p* < 0.0001). Post hoc Tukey’s test revealed significant differences between Group I and Group II (*p* = 0.0210), Group I and Group III (*p* < 0.0001), and between Group II and Group III (*p* = 0.0010). This indicates that the RPCs inside the disk varied significantly across all groups, with the highest values observed in keratoconic eyes after PKP.

In terms of RPC vessel density in the peripapillary superior region, Group I had a mean density of 53.45 ± 4.70 µm, Group II showed a similar mean density of 53.00 ± 4.88 µm, and Group III had a slightly higher mean density of 53.69 ± 4.61 µm. The ANOVA demonstrated a significant difference (*p* = 0.0005). Post hoc analysis revealed that the difference between Group I and Group III was highly significant (*p* = 0.0001), while the differences between the other groups were not statistically significant, indicating that control eyes had significantly higher RPC vessel density in the peripapillary superior region compared to keratoconic eyes without PKP.

For the RPC vessel density in the peripapillary inferior region, Group I recorded a mean of 52.35 ± 4.49 µm, Group II had a slightly lower mean of 51.87 ± 4.39 µm, and Group III had a mean of 52.61 ± 4.54 µm. The ANOVA showed a highly significant difference among the groups (*p* < 0.0001). Post hoc analysis revealed a significant difference between Group I and Group III (*p* = 0.0010), but not between the other groups, suggesting that the peripapillary inferior vessel density was significantly lower in keratoconic eyes after PKP compared to control eyes.

Regarding the RPC vessel density peripapillary average, Group I showed a mean of 52.92 ± 4.33 µm, Group II had a mean of 52.45 ± 4.30 µm, and Group III had a mean of 53.17 ± 4.35 µm. The ANOVA indicated a significant difference (*p* < 0.0001). Post hoc analysis demonstrated a significant difference between Group I and Group III (*p* = 0.0001), indicating that the peripapillary average vessel density was significantly higher in control eyes compared to keratoconic eyes without PKP.

Lastly, for the RPC whole vessel density, Group I had a mean of 54.38 ± 2.28 µm, Group II had a mean of 54.60 ± 2.33 µm, and Group III had a mean of 54.26 ± 2.26 µm. The ANOVA did not show any significant differences among the groups (*p* = 0.6179); therefore, no post hoc analysis was performed.

In summary, the analysis of RPC parameters revealed significant variations across the groups, particularly in the RPCs inside the disk and peripapillary vessel densities, where control eyes generally exhibited higher values compared to keratoconic eyes, especially those without PKP. These differences were most pronounced in the peripapillary superior and inferior regions and the peripapillary average vessel density, emphasizing the impact of keratoconus and PKP on retinal vascular structures.

### 3.3. Correlation and Multiple Linear Regression Analysis

**Figure 1:**
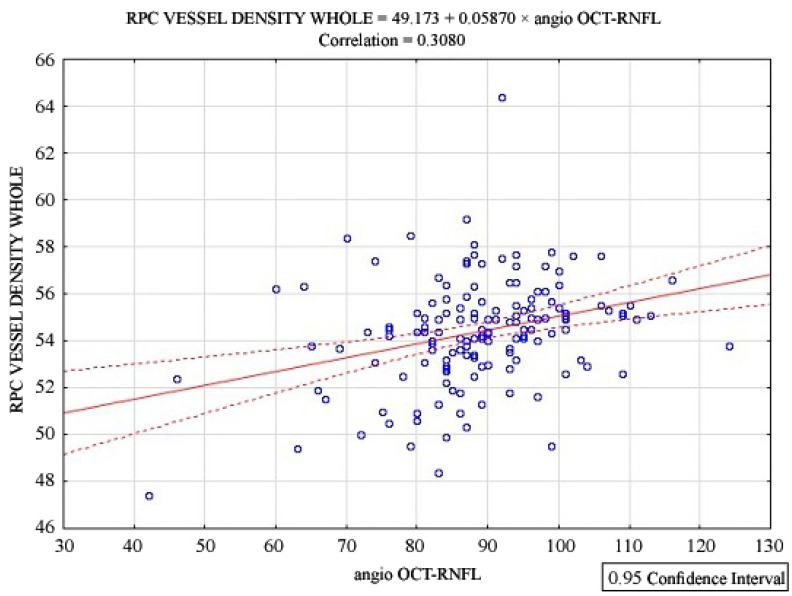
Positive, significant correlation for RPC whole vessel density with angio-OCT RNFL thickness.

Correlation analysis was performed to determine the relationship between the RPC vessel density and the RNFL thickness as measured by means of angio-OCT. The scatter plot in Figure 1 illustrates the relationship between the RPC vessel density (whole) and the angio-OCT RNFL thickness.

The analysis revealed a positive and significant correlation between the RPC vessel density and the RNFL thickness (r = 0.3080, *p* < 0.0001). This indicates that as the RNFL thickness increases, the RPC vessel density also tends to increase. The correlation coefficient (r = 0.3080) suggests a moderate correlation, meaning that while there is a relationship between these two parameters, it is not a strong one.

The regression line displayed on the scatter plot shows the linear relationship between RPC vessel density (whole) and angio-OCT RNFL thickness, with the equation RPC vessel density whole = 49.173 + 0.05870 × angio-OCT RNFL. The dashed lines around the regression line represent the 95% confidence interval, indicating the range within which we expect the true regression line to lie with 95% confidence.

This result suggests that changes in the RNFL thickness are moderately associated with changes in RPC vessel density, which may have implications for understanding the vascular aspects of diseases affecting the RNFL.

In turn, the regression analysis revealed that RNFL thickness had a significant positive association with RPC vessel density (β1 = 0.054, *p* < 0.001), indicating that an increase in RNFL thickness is associated with an increase in RPC vessel density. Axial length, on the other hand, had a negative association with RPC vessel density (β2 = −0.033, *p* = 0.021), suggesting that as axial length increases, the RPC vessel density decreases. The model explained a moderate proportion of the variance in RPC vessel density, with an R-squared value of 0.35, indicating that 35% of the variability in RPC vessel density could be accounted for by RNFL thickness and axial length.

## 4. Discussion

In keratoconus, changes can be observed not only in the anterior segment of the eye but also in the posterior segment. Pathologies in the posterior segment require further research. Angio-OCT helps in diagnosis and is currently widely used in clinical practice.

In our research, we evaluated different parameters, such as the average RNFL, GCC, and CRT, using OCT and angio-OCT in keratoconus patients after penetrating keratoplasty. We evaluated RPCs in detail, as they likely represent an important new parameter in the further diagnosis of this disease. We showed that most parameters were significantly different between patients with KC and control subjects.

To the best of our knowledge, no prior research has assessed these parameters in KC after PKP. We aimed to conduct this study on as many keratoconic eyes as possible, resulting in a study sample of 149 keratoconic eyes.

In our research, the RNFL was statistically significantly thinner in keratoconic eyes compared to controls. For the GCC, the measurements between groups were similar. The CRT was greater for the keratoconic group, and this difference was statistically significant. The results were similar when using OCT and angio-OCT.

Cankaya et al. [25] suggested that between patients with keratoconus and healthy participants, the RNFL is more comparable than ONH parameters. Other studies in the available literature have presented different results for RNFL, GCC, and CRT parameters. A study by Orman et al. [26] showed no statistically significant difference in macular thickness between KC and control groups, some decrease in the GCC in the keratoconus group, and a significant decrease in the thickness of the 5th and 11th h of the peripapillary RNFL. For the RNFL, they obtained results similar to those in our study. Additionally, Aydemir et al. [24] revealed that the RNFL in keratoconic eyes was thinner compared to that in astigmatic eyes and a control group. Uzunel et al. [25] showed that ganglion cell parameters, the RNFL, and macular thickness in all KC stages were lower than those in controls. On the other hand, Özsaygılı et al. [26] found that there were no significant differences in the RNFL between KC and control groups.

The results of these various studies could be influenced by the use of different devices, group size, and the fact that our study also included eyes after PKP.

Sahebjada et al. [27] showed that keratoconus patients had significantly greater mean retinal thickness in the central fovea and inner and outer macula and greater macular volume compared to the non-keratoconus group (*p* < 0.05). These findings are similar to our results, where the CRT was significantly greater in the KC groups. The authors also suggested that increased foveal thickness could relate to the retinomotor movements of the photoreceptors and their elongation as a compensation for the change in corneal curvature. They hypothesized that the retinal tissue grows to compensate for the thinning of the cornea and to prevent “disorganization” of the eye; however, this requires more research. Fard et al. [28] showed that the overall retinal thickness in KC patients was greater than that in a control group. Other studies found no differences in macular thickness between KC and control groups [29,30].

Most of our participants were myopic; therefore, their axis length was greater. This could also affect the CRT. Choi et al. [31] showed that as the level of myopia increases, the thickness of the fovea also increases; however, the thickness of the peripapillary RNFL decreases. Lim et al. [32] revealed that the fovea is thicker with myopia. These findings require further investigation.

Additionally, irregular astigmatism could have an impact on the results of OCT obtained in patients with keratoconus [33].

Previously, keratoconus was considered to be a noninflammatory disease. Now, however, it is becoming increasingly clear that the etiopathogenesis of this disease involves inflammation [34] and oxidative stress [35]. Increased stress-induced generation of reactive oxygen species, apoptosis in human keratoconus fibroblasts, and genetics [36] all play a role. One piece of evidence of the influence of inflammatory mediators in keratoconus is the increase in choroidal thickness [37,38]. Collagen is also disturbed in KC patients [39], which may result in disturbances in the structure of blood vessels. Kuivaniemi et al. [40] pointed out that the mutations in types I, II, III, IX, X, and XI collagens cause a wide spectrum of diseases of cartilages, bones, and blood vessels. Moreover, keratoconus often occurs with connective tissue diseases, such as Ehlers–Danlos and Marfan syndromes [41]. Connective tissue builds blood vessels in the human body; therefore, these diseases may damage and reduce the number of vessels in keratoconic eyes.

In many studies, vessel density was found to be lower in keratoconus groups. Leclaire et al. [42] noticed a reduced vessel density, especially in the superficial capillary plexus. Furthermore, they proposed a link between cardiovascular disease and KC due to microvascular changes seen in KC.

In our research, RPCs inside the disc were significantly decreased in keratoconic eyes compared to control eyes. Moreover, in each group (I, II), the results were significantly lower than those in the control group (*p* < 0.05).

The abovementioned study, which investigated a small group consisting of 26 keratoconic eyes, also showed significantly decreased RPCs inside the disc in the KC group (*p* = 0.0033) compared to healthy participants, whereas the RPC “whole” was similar in the two groups, as in our study. Dogan et al. [37] also showed that the density of RPCs inside the disc vessel was significantly decreased in the KC group compared to a control group (*p* < 0.001). In contrary to our study, the retinal nerve fiber layer thickness did not significantly differ between the two groups (*p* = 0.581). Their study sample consisted of 32 keratoconic eyes, and patients after corneal transplantation were excluded, whereas in our study, we investigated 149 keratoconic eyes, and 97 were after PKP. Similar to our results, their study found the retinal thickness in the fovea to be greater in KC patients than a control group, but this result was not statistically significant. Other studies have also shown decreased RPCs in KC patients [43,44].

An interesting relationship was revealed by Zhang et al. [45]. They investigated patients with Bietti Crystalline Dystrophy and revealed that the RPC density was significantly lower than that in a control group. They suggested that the retinal pigment epithelium (RPE) is damaged and lost in this disease. The RPE expresses vascular endothelial growth factor (VEGF). Lower VEGF levels may affect retinal vessel growth, leading to a decrease in the retinal capillary density. Aydemir et al. [24] showed that KC groups had significantly lower IPL (inner plexiform layer), ONL (outer nuclear layer), RPE, and ORL (outer retinal layer) thicknesses than did patients with astigmia and healthy controls (*p* < 0.05 for each). It is possible that lower VEGF in KC also causes decreased vessel density; however, this assumption requires further research.

Moreover, corneal transplantation could affect the RPC parameter. In our study, RPCs inside the disc were the lowest in keratoconic eyes after PKP. Positive vitreous pressure during penetrating keratoplasty could have an influence on vessels [46,47].

In addition, myopia could influence RPCs. Ye et al. [17] showed that RPCs and the peripapillary RNFL were decreased in myopic eyes compared to a control group. The patients in our study were mostly myopic, and the RNFL was significantly lower in keratoconic eyes. On the other hand, the CRT was greater in KC than the control group. La Spina et al. [48] showed that myopic eyes have lesser peripapillary blood flow and smaller vessel diameters.

This study has several limitations that should be acknowledged. First, the population studied was relatively homogeneous, consisting solely of Polish Caucasians without systemic diseases, which may limit the generalizability of the findings to other ethnic groups and populations. A more heterogeneous study population would enhance the applicability of the results to a broader demographic.

Second, the retrospective nature of the study imposes certain limitations on the data collected, including the potential for selection bias and the inability to control for all confounding variables. Prospective studies would be needed to confirm these findings and provide a more comprehensive understanding of the impact of keratoconus and penetrating keratoplasty PKP on retinal and ocular metrics.

Additionally, while this study highlighted significant findings regarding radial RPCs and their correlation with RNFL thickness post-PKP, further investigation is necessary to thoroughly explore the potential mechanisms underlying these observations. Specifically, this study did not fully address the lasting impacts of PKP on RPCs and other ocular metrics in patients with KC. Understanding the possible mechanisms causing alterations in RPC function and retinal thickness in KC patients post-PKP requires further detailed research.

Our results from a linear multiple regression analysis suggest that both the RNFL thickness and axial length are important predictors of RPC vessel density in keratoconus patients. The positive relationship between the RNFL thickness and RPC vessel density aligns with the understanding that a thicker RNFL may support a denser capillary network. The negative association with axial length highlights the influence of ocular elongation on retinal vasculature, potentially due to stretching effects that reduce capillary density. Clinically, these findings emphasize the need to consider the axial length when interpreting RPC vessel density in patients with varying degrees of myopia or other conditions that affect axial length. This highlights the importance of considering these factors in clinical assessments of retinal health and suggests that future studies should further investigate the mechanisms linking axial length with retinal vasculature. Understanding these relationships could improve diagnostic accuracy and patient management in conditions affecting the retina.

These limitations should be considered when interpreting the results, and future research should address these areas to provide a more robust understanding of the phenomena observed in this study.

The conclusions of this study highlight the potential of OCT as a valuable diagnostic tool in the management of keratoconus. However, to solidify its role in clinical practice, future research should focus on standardizing OCT diagnostic criteria and exploring the long-term outcomes of OCT-guided interventions in keratoconus patients. Additionally, there is a need to assess the cost-effectiveness of incorporating OCT into routine screening protocols, particularly in high-risk populations. By addressing these areas, the use of OCT in keratoconus diagnosis could be further refined and optimized, ultimately enhancing patient care. In current clinical practice, OCT and angio-OCT are increasingly employed as standard imaging techniques for evaluating retinal structures in various ocular conditions. Our study’s findings, particularly regarding the assessment of RPCs and the RNFL thickness, can readily be integrated into routine diagnostic and follow-up protocols for keratoconus patients. The reduction in RPC density in keratoconus patients post-PKP highlights the potential for angio-OCT to serve as a valuable tool for early detection of vascular alterations, thereby guiding timely interventions. These advancements in imaging technology allow for more precise monitoring of structural and vascular changes in both pre- and post-surgical keratoconus patients, aligning with current practice standards aimed at improving patient outcomes through enhanced diagnostic accuracy and management strategies.

## 5. Conclusions

This study explored the RPC parameters in keratoconus patients who had undergone PKP and found that the density of RPCs inside the disc was significantly reduced in these patients. The lowest RPC values were observed in eyes post-PKP. Additionally, we discovered that only the RPC whole vessel density showed a significant correlation with the RNFL thickness as measured with angio-OCT. This suggests that the RPC parameter, particularly RPCs inside the disc, could be a promising new diagnostic tool for further assessing keratoconus. Further investigation is required to fully explore the potential mechanisms underlying the observed alterations in RPC function and retinal thickness in KC patients post-PKP. Understanding these mechanisms could lead to better clinical management and treatment outcomes for keratoconus patients.

Finally, while this study identified important correlations, the complex interactions between corneal transplantation, RPC parameters, and other ocular health metrics remain underexplored. Future research should aim to elucidate these relationships and consider the long-term effects of PKP on these parameters to better inform clinical practice.

## Figures and Tables

**Table 1:** OCT and angio-OCT parameters in particular groups.

OCT/Angio-OCT	Parameter [μm]	Group	*n*	Mean ± SD	95%Cl	Median	*p*-Value (ANOVA)	*p*-Value (Post Hoc Tukey’s Test)
OCT	RNFL	Group I	52	88.96 ± 13.02	85.34–92.59	89.50	0.0037	0.9820 ^1^
		Group II	97	88.33 ± 10.95	86.12–90.54	88.00		0.1780 ^2^
		Group III	144	92.42 ± 7.67	91.15–93.68	92.00		0.0115 ^3^
	GCC	Group I	52	101.81 ± 8.77	99.37–104.25	103.00	0.2027	-
		Group II	97	100.78 ± 9.40	98.89–102.68	100.00		
		Group III	144	102.81 ± 7.52	101.57–104.05	102.50		
	CRT	Group I	52	269.85 ± 18.99	264.56–275.13	271.00	0.0016	0.0340 ^1^
		Group II	97	267.90 ± 19.21	264.02–271.77	268.00		0.8745 ^2^
		Group III	144	260.00 ± 21.14	256.52–263.48	254.50		0.0175 ^3^
Angio-OCT	RNFL	Group I	52	88.96 ± 13.45	92.71–90.00	90.00	0.0028	0.9820 ^1^
		Group II	97	88.60 ± 11.18	90.85–88.00	88.00		0.1262 ^2^
		Group III	144	92.85 ± 7.92	94.16–93.00	93.00		0.0103 ^3^
	GCC	Group I	52	101.92 ± 9.30	104.51–102.00	102.00	0.1351	-
		Group II	97	101.21 ± 9.23	103.07–101.00	101.00		
		Group III	144	103.40 ± 7.70	104.66–103.00	103.00		
	CRT	Group I	52	270.56 ± 20.65	276.31–274.00	274.00	0.0209	0.5367 ^1^
		Group II	97	266.89 ± 19.17	270.75–267.00	267.00		0.0248 ^2^
		Group III	144	262.09 ± 20.47	265.46–257.00	257.00		0.1635 ^3^

Group I—keratoconic eyes without PKP, Group II—keratoconic eyes after PKP, Group III—control eyes. RNFL—retinal nerve fiber layer, GCC—ganglion cell complex, CRT—central retinal thickness, 95%Cl—95% confidence interval, *n*—number of eyes, OCT—optical coherence tomography, Angio-OCT—optical coherence tomography angiography. ^1^ *p*-value (group I vs. group II); ^2^ *p*-value (group I vs. group III); ^3^ *p*-value (group II vs. group III).

**Table 2:** RPC parameters in particular groups.

Parameter [μm]	Group	*n*	Mean ± SD	95%Cl	Median	*p*-Value (ANOVA)	*p*-Value (Post Hoc Tukey’s Test)
RPCS inside disk	Group I	52	42.23 ± 7.22	42.23–46.49	41.80	0.0000	0.0210 ^1^
	Group II	97	44.36 ± 7.65	39.73–42.45	44.70		0.0000 ^2^
	Group III	144	41.09 ± 6.75	48.65–50.39	41.10		0.0010 ^3^
RPC vessel density peripapillary superior	Group I	52	53.45 ± 4.70	51.64–54.35	53.40	0.0005	0.5977 ^1^
							0.0001 ^2^
							0.0623 ^3^
	Group II	97	53.00 ± 4.88	52.76–54.62	52.50	-	-
	Group III	144	53.69 ± 4.61	51.02–51.72	53.60		
RPC vessel density peripapillary inferior	Group I	52	52.35 ± 4.49	50.64–53.09	52.10	0.0000	0.5457 ^1^
	Group II	97	51.87 ± 4.39	51.69–53.52	50.95		0.0010 ^2^
	Group III	144	52.61 ± 4.54	50.39–51.15	52.90		0.2608 ^3^
RPC vessel density peripapillary average	Group I	52	52.92 ± 4.33	51.26–53.65	52.60	0.0000	0.5234 ^1^
	Group II	97	52.45 ± 4.30	52.30–54.05	51.80		0.0001 ^2^
	Group III	144	53.17 ± 4.35	50.82–51.48	53.00		0.1216 ^3^
RPC vessel density whole	Group I	52	54.38 ± 2.28	53.95–55.25	54.50	0.6179	-
	Group II	97	54.60 ± 2.33	53.81–54.72	54.65		
	Group III	144	54.26 ± 2.26	42.23–46.49	54.40		

Group I—keratoconic eyes without PKP, Group II—keratoconic eyes after PKP, Group III—control eyes. 95%Cl—95% confidence interval, *n*—number of eyes. ^1^ *p*-value (group I vs. group II); ^2^ *p*-value (group I vs. group III); ^3^ *p*-value (group II vs. group III).

## Data Availability

The data used to support the findings of this study are included in the article. The data cannot be shared due to third-party rights and commercial confidentiality.
